# Circulating Immunosuppressive Regulatory T Cells Predict Risk of Incident Cutaneous Squamous Cell Carcinoma

**DOI:** 10.3389/fmed.2021.735585

**Published:** 2021-11-02

**Authors:** Dana E. Rollison, Jane L. Messina, Basil S. Cherpelis, Neil A. Fenske, Michael J. Schell, Dennis O. Adeegbe, Yayi Zhao, Rossybelle P. Amorrortu, Afua A. Akuffo, Rebecca S. Hesterberg, Pearlie K. Epling-Burnette

**Affiliations:** ^1^Department of Cancer Epidemiology, Moffitt Cancer Center, Tampa, FL, United States; ^2^Departments of Pathology and Cutaneous Oncology, Moffitt Cancer Center, Tampa, FL, United States; ^3^Department of Dermatology and Cutaneous Surgery, University of South Florida College of Medicine, Tampa, FL, United States; ^4^Department of Biostatistics and Bioinformatics, Moffitt Cancer Center, Tampa, FL, United States; ^5^Department of Immunology, Moffitt Cancer Center, Tampa, FL, United States

**Keywords:** epidemiology, regulatory T cells, ultraviolet radiation exposure, cutaneous squamous cell carcinoma, risk stratification

## Abstract

Ultraviolet radiation exposure (UVR) is a risk factor for cutaneous squamous cell carcinoma (cuSCC) and has been shown to be positively associated with circulating immunosuppressive regulatory T cells (“Tregs”). However, the risk of cuSCC in association with circulating Tregs has not been studied. The aim of this study was to determine whether circulating Treg levels are associated with cuSCC development, particularly in the context of high UVR. Blood and spectrophotometer-based UVR measurements were obtained on 327 immunocompetent individuals undergoing routine skin cancer screenings at baseline and followed for up to 4 years for incident cuSCC development within a prospective cohort study. Proportions of phenotypically distinct Tregs, especially CCR4^hi^ and CLA^+^ cells which are associated with activation and homing, respectively, were measured by flow cytometry. Tregs in cuSCC tumors were assessed using immunohistochemistry and graded for solar elastosis, a measure of cumulative UVR damage. Of several Treg phenotypes examined, higher levels of circulating CCR4^hi^ Tregs at baseline were significantly associated with increased risk of subsequent cuSCC; those with higher levels of both CCR4^hi^ and UVR were four times more likely to develop cuSCC compared to those with lower levels of both (Hazard Ratio = 4.11, 95% CI = 1.22–13.90). Within cuSCC tumors, CCR4^hi^ Tregs were positively associated with solar elastosis. Results show that a higher proportion of CCR4^hi^ peripheral Tregs predicts incident cuSCC up to 4 years, especially among highly UV-exposed individuals. Research of the underpinning biology of Tregs in UVR-associated skin damage may possibly reveal novel opportunities for screening, prevention, and treatment.

## Introduction

Keratinocyte carcinomas (KC) including cutaneous squamous cell carcinoma (cuSCC) and basal cell carcinoma (BCC) are the most commonly diagnosed cancers in the U.S. (1–3), with more than five million KC cases diagnosed each year (4). CuSCC typically comprised 20% of KC (1, 5) however trends are increasing with a recent study reporting a 1:1 ratio of cuSCC to BCC in adults ages 65+ (4). CuSCC is more often aggressive than BCC, with 4,000 people dying from invasive cuSCC in the U.S. each year (6). Ultraviolet (UV) radiation exposure (UVR) is the major environmental risk factor for KC, and chronic lifetime UVR has been associated specifically with cuSCC risk (2, 7–9). While chronic lifetime UVR results in the accumulation of UVR-induced DNA damage (9), leading to skin carcinogenesis (10, 11), UVR may also contribute to cuSCC development through an immunosuppressive pathway. Immunosuppression is a well-established risk factor for cuSCC, with heart, kidney, blood, and marrow transplant patients experiencing 16- to 68- fold risks of cuSCC compared to the general population (12, 13). Furthermore, long term use of oral corticosteroids has been associated with increased cuSCC risk among immunocompetent individuals (14). While mouse studies have shown that UVR is immunosuppressive (15, 16), no epidemiologic studies have sought to measure the interplay between UVR and immune function in relation to cancer etiology in humans.

UVR can lead to systemic immune suppression as well as antigen-specific immune tolerance due to the induction of regulatory T cells (“Tregs”) which are known to have immunosuppressive effects (16, 17). Specifically, in mice, thymically-derived Tregs increase following exposure to low doses of UVR in the absence of tumors (18). In humans, clinical studies in patients with multiple sclerosis and psoriasis have shown that circulating Tregs are induced after treatment with UV phototherapy (15, 19, 20). The expansion of Tregs in circulation is mediated by antigen activation which enables their suppressive mechanisms and can trigger homing into the skin (16, 18). Studies have identified subpopulations of Tregs that display specific markers associated with skin homing, including the carbohydrate epitope cutaneous lymphocyte-associated antigen (CLA) that binds E-selectin and initiates tethering and rolling along cutaneous venules (18, 21, 22). Compared to Tregs in circulation, tumor-infiltrating Tregs contain an activated population responsive to cognate self-antigen capable of eliciting immune suppression (23). Unlike naïve Tregs that express the molecules CD45RA and CD27 (Naïve), activated Tregs downregulate both of these surface molecules (24, 25) and induce the expression of C-C chemokine receptor 4 (CCR4) (26) that also contributes to skin trafficking along with expression of CLA (27, 28).

We previously observed that recent UVR was positively correlated with CD45RA^−^/CD27^−^ activated Tregs, CLA^+^ and CCR4^hi^ Tregs in a cross-sectional analysis of baseline samples from a subset of skin cancer screening patients participating in a prospective cohort study of skin cancer in Tampa, FL, the Viruses in Skin Cancer (VIRUSCAN) Study (29). To determine whether UVR and circulating Tregs at baseline were associated with subsequent diagnosis of cuSCC, we conducted a prospective analysis among the same VIRUSCAN participants.

## Materials and Methods

### Study Population

The VIRUSCAN Study population and methods have been described in detail previously (30). Briefly, individuals age 60+ undergoing routine skin cancer screening exams at the University of South Florida (USF) Dermatology Clinic were enrolled between July 2014 and August 2017 and followed for subsequent KC diagnosis through September 2018. Four hundred twenty-one participants were enrolled in the first year and confirmed not to have a prevalent KC at enrollment. Of these, 327 returned for at least one follow-up visit, had available circulating Treg flow cytometry results and were included in the current analysis. Four participants were missing baseline spectrophotometer readings and were excluded from the analyses related to UVR. One participant was missing self-reported sun sensitivity and was excluded from the full Cox regression model of cuSCC. This study was approved by the USF Institutional Review Board and all patients provided written informed consent.

### Skin Cancer Risk Factor and UVR Exposure Measurement

Study participants completed an electronic questionnaire at baseline including information on demographics, skin cancer risk factors, and medical history (30). A Konica Minolta CM-600D spectrophotometer with SpectraMagic NX Lite USB Ver.25 software was used to obtain quantitative and objective measurements of recent UVR exposure at baseline. Specifically, spectrophotometer readings of skin pigmentation were obtained from the sun-unexposed inner upper arm (i.e., the axilla) and sun-exposed forearm. The degree of recent tanning in response to recent UVR exposure was measured by calculating the difference between the pigment readings on the forearm and the axilla using the Commission Internationale de I'Éclair age (CIE) L^*^a^*^b^*^ system and CIE76 formula: ΔE^*^ab = [(ΔL^*^)^2^ + (Δa^*^)^2^ + (Δb^*^)^2^]^1/2^ (31). This measurement has been validated as a quantitative marker of UVR exposure experienced in the past week (30). Additionally, the brightness measure L^*^ obtained from the unexposed axilla was previously reported to be associated with the melanin index (32), and was found to be associated with self-reported untanned skin color among VIRUSCAN Study participants (30).

### Measurement of T Regulatory Cells in Circulation

Blood samples were obtained at baseline and Peripheral Blood Mononuclear Cells (PBMCs) were isolated and frozen for future use. PBMCs were thawed, and proportions of circulating Tregs were determined by flow cytometry analysis, as described previously (29). Flow cytometry assays were performed using PBMCs with data acquired on an LSR II flow cytometer (BD Biosciences) and analyzed using FlowJo v9 (FlowJo, RRID:SCR_008520, BD Bioscience) to examine the proportions of CD8^+^ and CD4^+^ T cells, total Tregs and Treg subpopulations as defined in Supplementary Figure 1, and as previously described (29).

### Ascertainment of Incident cuSCC

Study follow-up visits were integrated into routine clinical care with total body skin exams for skin cancer screening occurring every 6–12 months. During these exams, the entire integument was examined for suspicious lesions, including the scalp, underarms, groin, and buttocks. When indurated cutaneous lesions suspected to be malignant were identified, they were biopsied, formalin-fixed, and sent for pathology review per standard clinical protocol. Study coordinators reviewed pathology reports to identify incident cases of KC diagnosed among cohort participants. Clinical characteristics of the KC tumors were recorded, including date and stage of diagnosis, anatomic location of the tumor, and treatment received. Participants were considered cuSCC cases if they were not diagnosed with KC at baseline and developed at least 1 incident cuSCC, basosquamous, or SCC/BCC merged tumor during the follow-up period. Participants who were not diagnosed with the above described lesions were considered to be non-cases. Person-time for incident cuSCC cases was calculated as the number of days between study enrollment and time when the first incident cuSCC was biopsied, while the person-time for non-case participants was calculated as the number of days between enrollment and the last total body skin exam.

### Measurement of T-Regulatory Cells and Solar Elastosis in Tumor Tissues

Formalin fixed paraffin embedded (FFPE) tumor tissue recuts were obtained from the USF Pathology department for 8 cuSCC tumors identified from 7 participants during the first year of follow-up. To augment the sample size for the tumor analysis, recuts of 16 cuSCC tumors diagnosed at the time of study enrollment were also included. These recuts were from 12 participants who were excluded from the main prospective analysis. The study dermatopathologist confirmed all cuSCC diagnoses and graded the degree of solar elastosis present in the adjacent normal tissue, a measure of cumulative UVR (30). Solar elastosis was graded on a 4-point scale: 0: no presence of elastotic fibers or very occasional elastotic fibers; 1: scattered elastotic fibers lying as individual units between collagen bundles; 2: densely scattered elastotic fibers distributed predominantly as bushels rather than individual units; 3: dense aggregates of elastotic fibers forming amorphous deposits of blue-gray material with lost fiber texture (33–35).

The cuSCC FFPE tumor recuts were mounted on glass slides and underwent immunohistochemistry (IHC) for CD3/FoxP3 and CCR4/FoxP3 using a two-color procedure. All slides were stained using a Ventana Discovery XT automated system (Ventana Medical Systems, Tucson, AZ) with proprietary reagents. Slides were deparaffinized on the automated system with EZ Prep solution. Heat-induced antigen retrieval using Cell Conditioning 1 was used for all stains. For the FoxP3/CCR4 two-color stain, the mouse primary antibody to Foxp3 (Abcam Cat# ab20034, RRID:AB_445284, clone 236A/E7, 1:400) and the rabbit primary antibody to CCR4 (LS Bioscience, Seattle, WA, 1:800, discontinued since staining was performed) were incubated for 28 min. The anti-mouse secondary antibody was incubated for 4 min and visualized with the Ventana anti-rabbit diaminobenzidine (DAB) multimeter; the anti-rabbit secondary antibody was incubated for 16 min and visualized with alkaline phosphatase. For the CD3/FoxP3 double stain, the mouse primary antibody to CD3 (prediluted, Ventana) and the rabbit primary antibody to FoxP3 (Abcam Cat# ab20034, RRID:AB_445284, 1:25) were incubated for 60 min. The anti-mouse secondary antibody was incubated for 4 min and visualized with DAB and the anti-rabbit secondary antibody was incubated for 4 min and visualized with alkaline phosphatase. For all stains, the detection system used was the Ventana OmniMap kit and slides were counterstained with hematoxylin, dehydrated, and coverslipped.

The IHC stained slides were imaged using an Aperio AT2 whole slide scanner (Leica Biosystems Inc., Vista, California) with a 20X 0.7NA objective lens. The whole slide images were viewed using the Aperio Imagescope software (RRID:SCR_014311) and identical sized rectangular regions of interest (ROIs) were annotated under the guidance of the study pathologist, focusing on areas of greatest inflammation. These ROIs were extracted into TIFF format images and imported into Definiens Tissue Studio image analysis software (Definiens AG, Munich, Germany) where cell and nucleus detection algorithms were used to enumerate the number of positive cell and negative cells for each marker. Identical intensity thresholds for cell positivity were used for all images stained with the same marker and confirmed by study pathologist.

### Statistical Methods

Participants' demographic and other baseline characteristics were described separately for patients who developed incident cuSCC and those who did not. Log-rank *p*-values were calculated to compare the time-to-incidence between categories of each variable. To understand the effect of UVR on the development of incident cuSCC, the cumulative incidence of cuSCC was plotted, stratified by tertiles of UVR as determined using spectrophotometer data obtained from all participants who returned to the clinic at least once. Age- and sex-adjusted Cox proportional hazard ratios (HR) and 95% confidence intervals (95% CI) were calculated to estimate the associations between UVR levels in the middle and highest tertiles vs. the lowest tertile and the risk of incident cuSCC. Based on similarity of the incidence curves and the estimated HR's, the categories of the middle and high tertiles of UVR were combined and compared to the lowest tertile.

Given limited pre-existing knowledge regarding the clinical utility of Treg cut points, the levels of each Treg subpopulation were categorized into three categories using the reduced monotonic regression method (36) to differentiate between the cuSCC risk groups. This method maximizes the difference in predicted risk, while maintaining the sample size of each category at ≥10%. Resulting cut points for categorizing higher, medium and lower Tregs within each subpopulation defined were as follows: 81.7 and 88.9 for CCR4^hi^ Tregs, 59.4 and 66.2 for CLA^+^ Tregs, and 8.05 and 13.7 for CD45RA^−^/CD27^−^ Tregs. Cumulative incidence of cuSCC was plotted for each Treg category (Supplementary Figure 2), and corresponding age- and sex-adjusted Cox regression HR's and 95% CI's were calculated to estimate the association between Treg levels in the middle and highest categories vs. the lowest category and the risk of incident cuSCC. For each Treg subpopulation, the categories of the middle and high groups of Treg levels were combined and compared to the lowest level, based on similarity of the incidence curves and the estimated HR's. Subsequently, the cumulative incidence curve for cuSCC was plotted by higher vs. lower circulating Tregs, separately for CCR4^hi^ Treg, CLA^+^ Treg, and CD45RA^−^/CD27^−^ Tregs. To account for potential confounding effects, a stepwise elimination process was implemented including the following variables: age, sex, recent UVR, job working under the sun, sun sensitivity, self-reported skin color, spectrophotometer-measured skin color, hair color, number of moles on body, and history of KC, along with the measurements of circulating Tregs. The variables retained (age, sex, and sun sensitivity) were included in the full Cox regression model. Cox regression HR and 95% CI were calculated to estimate the association between higher vs. lower circulating Tregs and the risk of incident cuSCC using both the age- and sex-adjusted model and the full model, separately for each Treg subpopulation.

Treg subpopulations that were significantly associated with incident cuSCC advanced to the next stage, where a joint effects analysis was conducted between UVR and each of the Treg phenotypes. Categorical variables representing different combinations of UVR levels and circulating Treg levels were created by grouping participants into those with lower CCR4hi Treg and lower UVR exposure level (reference group), higher CCR4hi Treg and lower UVR, lower CCR4hi Treg and higher UVR, and higher CCR4hi Treg and higher UVR. Cox models were then used to estimate the association between having both higher UVR and higher circulating Treg and the development of cuSCC. To assess whether the proportional hazard assumption was upheld for each model, the time-dependent coefficient was plotted across time of follow-up, followed by a formal score test to determine if the slope of the time-dependent coefficient deviated significantly from 0. In the single case of assumption violation (CLA^+^ Tregs in association with cuSCC), the data were restricted to the first 3 years during which the test for the proportional hazards assumption (37) was upheld. Model fitness was compared using analysis of deviance.

To examine the association between presence of Tregs in 24 cuSCC tumor tissues and solar elastosis in the adjacent normal tissue, the mean and standard deviation for each IHC-measured tumor T cell subpopulation was reported for each level of solar elastosis. The association between each tumor T cell subpopulation and increasing levels of solar elastosis was calculated using the Jonckheere-Terpstra trend test.

All analyses were conducted using R Statistical package, version 3.5.0 (R Foundation for Statistical Computing, Vienna, Austria).

## Results

Baseline characteristics of the participants enrolled in the VIRUSCAN cohort are described in Table 1, separately for those who subsequently developed cuSCC and those who did not. There were no significant differences between the two groups for most of the factors assessed, including age, sex, natural hair color, number of moles on the body, natural skin tone, ever having had a job working in the sun or history of a KC. The spectrophotometer-based measurements of sun-unexposed underarm skin also did not significantly differ between the two groups. However, self-reported lighter skin tone and a severe sunburn in reaction to 1 h of sun exposure were both significantly more common among those who subsequently developed cuSCC than those that did not (Table 1).

**Table 1:** Baseline characteristics of 327 skin cancer screening patients enrolled in year 1 of the VIRUSCAN Study compared between those that subsequently developed cutaneous squamous cell carcinoma (cuSCC) and those that did not.

**Baseline characteristics**	**cuSCC incidence**		
	**Non-case *n* = 271**	**Case *n* = 56**	***p*** **-value^a^**
**Age in years**			
Mean (SD)	69.2 (6.1)	70.2 (6.2)	
**Sex**			
Female	144 (53.1)	27 (48.2)	0.462
Male	127 (46.9)	29 (51.8)	
**Ever had a job working in the sun**			
No	194 (71.6)	43 (76.8)	0.739
Yes	77 (28.4)	13 (23.2)	
**Reaction to 1 h sun exposure**			
No change	64 (23.7)	10 (17.9)	0.045
Mild sunburn	123 (45.6)	20 (35.7)	
Severe sunburn with/without blister	83 (30.7)	26 (46.4)	
**Self-reported untanned skin color**			
Level 1	57 (23.3)	18 (36.7)	0.028
Level 2	122 (49.8)	23 (46.9)	
Level 3–10	66 (26.9)	8 (16.3)	
**Natural hair color**			
Blonde/Red	54 (20.1)	14 (25.9)	0.716
Light and medium brown	131 (48.7)	25 (46.3)	
Dark brown/Black	84 (31.2)	15 (27.8)	
**Moles on body**			
None (no moles)	78 (29.1)	20 (36.4)	0.536
<10 moles	133 (49.6)	26 (47.3)	
10–25 moles	36 (13.4)	7 (12.7)	
More than 25 moles	21 (7.8)	2 (3.6)	
**UVR exposure measured by spectrophotometer in tertiles** ^**b**^			
T1: (0.71, 10.4)	90 (33.7)	12 (21.4)	0.269
T2: (10.4, 15.5)	98 (36.7)	25 (44.6)	
T3: (15.5, 30.9)	79 (29.6)	19 (33.9)	
**Underarm spectrophotometer reading in tertiles** ^**c**^			
Lighter: (69.7, 81.1)	78 (29.2)	11 (19.6)	0.222
Middle: (67.3, 69.7)	98 (36.7)	20 (35.7)	
Darker: (37.5, 67.3)	91 (34.1)	25 (44.6)	
**History of keratinocyte carcinoma (KC) at study enrollment**			
No known KC	190 (70.1)	32 (57.1)	0.283
cuSCC only	31 (11.4)	9 (16.1)	
Basal cell carcinoma (BCC) only	42 (15.5)	14 (25.0)	
Both cuSCC and BCC	8 (3.0)	1 (1.8)	

a *P-values calculated using global log-rank test*.

*Tertiles of ^b^UVR exposure measurement and ^c^underarm spectrophotometer readings were determined using spectrophotometer data obtained from all VIRUSCAN Study participants who returned to the clinic at least once. The tertile calculation did not restrict to include only participants enrolled in the first year of the study*.

Among the 327 cohort participants, median follow-up time was 1,083 days, and the total follow-up time observed was 816 person-years. A total of 56 participants were diagnosed with at least one incident cuSCC during follow-up, corresponding to a cuSCC incidence rate of 6.86 per 100 person-years. Supplementary Figure 3 depicts the cumulative incidence rates for cuSCC by categories of recent UVR. The cumulative incidence of cuSCC was similar for participants whose recent UVR was in the middle and highest tertiles (Supplementary Figure 3A). When these two groups were combined and compared to those in the lowest tertile of recent UVR, a 74% increased risk of cuSCC associated with higher vs. lower recent UVR was observed, although not statistically significant (HR = 1.74, 95% CI = 0.87–3.49) (Supplementary Figure 3B).

**Figure 1:**
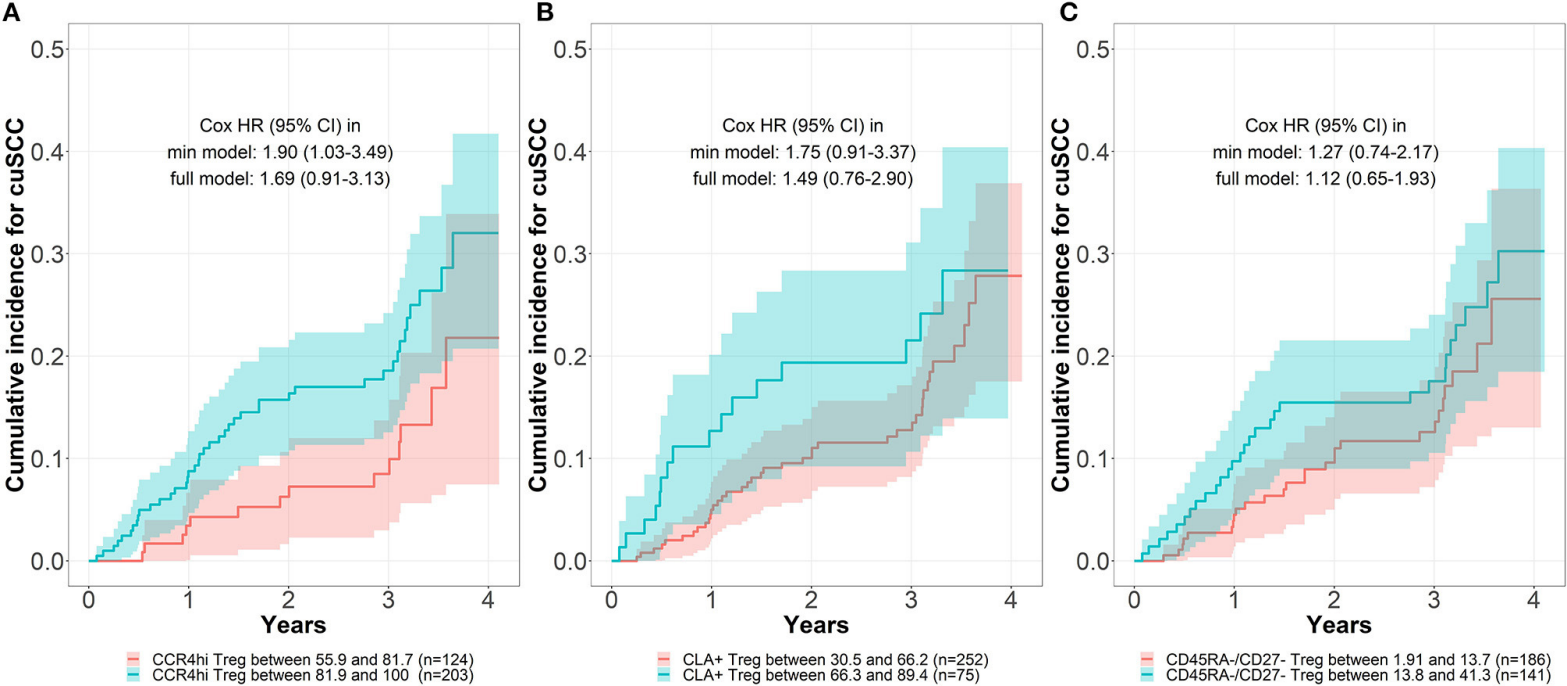
Cumulative incidence for cutaneous squamous cell carcinoma (cuSCC) by baseline levels of circulating Tregs. The cumulative incidence for cuSCC stratified by levels of baseline circulating Treg, separately for CCR4hi Treg **(A)**, CLA^+^ Treg **(B)**, and CD45RA^−^/CD27^−^ Treg cells **(C)**, as determined by the reduced monotonic regression in combination with visual inspection of the cumulative incidence curves. Hazard ratios (HR) and 95% confidence intervals (CI) were calculated using Cox proportional hazard models. The minimum (min) model was adjusted for age and sex, and the full model was further adjusted for self-reported skin reaction to 1 h of sun exposure.

Incidence of cuSCC was plotted for higher vs. lower circulating levels of CCR4^hi^, CLA^+^, and CD45RA^−^/CD27^−^ Tregs in Figure 1. Participants with higher circulating levels of these phenotypic subsets of Tregs assessed at baseline experienced higher incidence of cuSCC compared to those with lower levels, with the greatest separation of incidence curves observed for CCR4^hi^ Tregs (Figure 1A). The differences in cuSCC incidence between higher and lower levels of CLA^+^ Tregs was most pronounced within the first 3 years of follow-up, with the cumulative incidence curves converging after year three. The proportional hazards assumption was violated for CLA^+^ Tregs thus, the HR for CLA^+^ Tregs was calculated for the first 3 years of follow up only.

After adjusting for age and sex, cohort participants with higher levels of CCR4^hi^ Tregs in circulation at baseline experienced a statistically significant 90% increased risk of subsequent cuSCC compared to those with lower levels of CCR4^hi^ Tregs in circulation at baseline (Figure 1A: HR = 1.90, 95% CI = 1.03–3.49). After further adjustment for self-reported skin's reaction to 1 h of sun exposure, the hazard ratio remained elevated but was no longer statistically significant (HR = 1.69, 95% CI = 0.91–3.13). Cohort participants with higher levels of circulating CLA^+^ Tregs at baseline also experienced an increased risk of subsequent cuSCC, although this risk was not statistically significant with adjustment for age and sex (HR = 1.75, 95% CI = 0.91–3.37) or after further adjustment for skin's reaction to sun exposure. There was no increased risk of cuSCC associated with higher levels of circulating CD45RA^−^/CD27^−^ Tregs at baseline. Addition of CLA^+^ Treg to the existing CCR4hi Treg model did not significantly improve the model fitness (*p* = 0.77).

**Figure 2:**
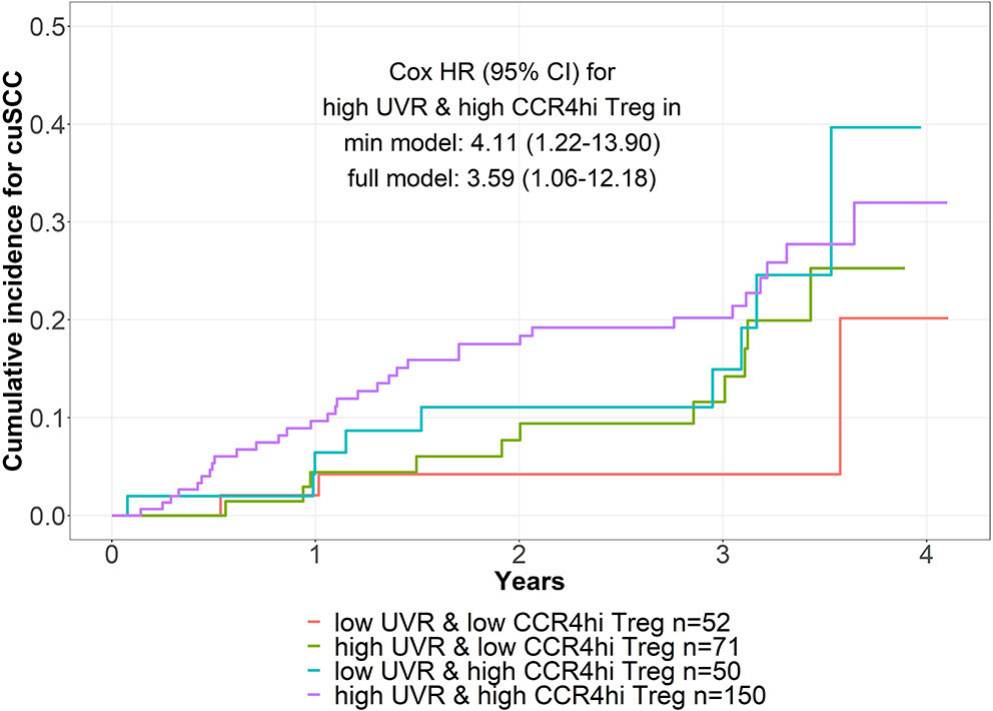
Joint effects of CCR4^hi^ Treg and spectrophotometer measured UV exposure (UVR) on risk of incident cutaneous squamous cell carcinoma (cuSCC). Cumulative incidence of cuSCC incidence among four groups of cohort participants defined by combinations of higher vs. lower UVR and higher vs. lower CCR4^hi^ Tregs at baseline. The minimum (min) model was adjusted for age and sex, and the full model was further adjusted for self-reported skin reaction to 1 h of sun exposure.

Figure 2 depicts the cuSCC incidence among four groups of cohort participants defined by combinations of higher vs. lower UVR and higher vs. lower enrichment of CCR4^hi^ Tregs at baseline. The highest incidence of cuSCC was observed in those with higher recent UVR and higher levels of CCR4^hi^ Tregs, with the risk of cuSCC being four times that of those with lower UVR and lower levels of CCR4^hi^ Tregs (HR = 4.11, 95% CI = 1.22–13.90), an association that remained statistically significant after further adjustment for self-reported skin reaction to the sun (HR = 3.56, 95% CI = 1.06–12.18). Supplementary Figure 4 shows illustrative examples of the solar elastosis grading and corresponding IHC staining performed on the cuSCC tumors. Among the 24 cuSCC tumors investigated by IHC staining, higher levels of solar elastosis (i.e., cumulative sun damage) measured in the adjacent normal tissue were significantly associated with higher proportions of CCR4-expressing T cells, as well as CCR4 and FoxP3 co-expressing Tregs in the tumor (Table 2).

**Table 2:** Associations between T cell subpopulations identified in incident cutaneous squamous cell carcinoma (cuSCC) and levels of solar elastosis in the normal tissue adjacent to tumor.

**Mean number of positively staining cells/sq mm**	**Solar elastosis^a^**			
	**Level 1 (*n* = 8)**	**Level 2 (*n* = 6)**	**Level 3 (*n* = 10)**	***p*** **-trend^2^**
	**Mean (SD)**	**Mean (SD)**	**Mean (SD)**	
CD3	16.1 (18.3)	22.2 (15.5)	12.9 (9.1)	0.772
FOXP3	54.5 (28.5)	66.3 (8.0)	65.3 (12.1)	0.895
CCR4	2.3 (2.9)	5.1 (7.9)	10.5 (8.9)	0.043
FOXP3 and CD3	12.5 (14.6)	18.2 (15.0)	11.2 (8.1)	0.693
FOXP3 and CCR4	2.2 (2.7)	4.3 (5.9)	8.6 (7)	0.043

a *None of the cuSCC tumors were graded as a solar elastosis level of 0, thus the category was not included in the table*.

In summary, while controlling for factors including age, gender, and skin sensitivity to sun exposure, participants with higher levels of circulating CCR4hi Tregs at baseline had a higher risk of developing cuSCC up to 4 years later. The risk of developing cuSCC was especially elevated among participants who had both higher levels of circulating CCR4hi Tregs and higher levels of recent UVR exposure. In addition, CCR4hi Tregs were more likely to be present in cuSCC tumors arising in areas of the skin that showed evidence of cumulative sun damage.

## Discussion

Our previous cross-sectional analysis demonstrated that higher levels of UVR, a well-documented risk factor for KC, were significantly associated with higher proportions of defined circulating Treg subpopulations, but not with total Tregs (29). In the current study with prospective monitoring for up to 4 years, individuals with higher circulating CCR4^hi^ Tregs and higher levels of UVR measured by a spectrophotometer were approximately four times more likely to develop cuSCC compared to those with lower CCR4^hi^ Tregs and lower UVR. Furthermore, the presence of Tregs expressing CCR4 in cuSCC tumors was associated with the presence of solar elastosis (cumulative sun damage) in the adjacent normal tissue was verified in a subset of patients. UVR may contribute to carcinogenesis by promoting skin infiltration associated with tissue remodeling that is induced through UVR-induced skin damage. We have shown previously that subpopulations of Tregs differ by age, sex, and race with lighter skinned individuals demonstrating higher levels of circulating activated Tregs at baseline associated with higher UVR (29). Adjustment for these factors demonstrated the independent contribution of circulating activated CCR4^hi^ Tregs as a predictor of cuSCC. Of note, there was no evidence that natural skin tone modified the association of activated Tregs and cuSCC (HR_lighter_ = 1.86, 95% CI = 0.74–4.69 vs. HR_darker_ = 1.83, 95% CI = 0.81–4.21, *P*_interaction_ = 0.92).

In immunocompetent hosts, developing neoplasms are shaped by factors present in the local microenvironment. While this process has been shown using elegant mouse models, factors leading to the permissive growth of naturally arising human tumors is poorly understood. At the time of detection, Tregs are frequently expanded in cancer patients including breast (38), pancreatic (39, 40), colon (41), non-small cell lung (42, 43) as well as hematological cancers (44–47). The majority of peripheral Tregs in healthy individuals typically display a CD3^+^CD4^+^FOXP3^+^CD25^+^CD127^dim^CD27^+^CD45RA^+^ phenotype with changes noted in the phenotype during activation (29, 45, 48). High levels of chemokine receptor CCR4 on Tregs in peripheral blood have been associated with the presence of active rheumatoid arthritis, with levels of CCR4 on Tregs positively correlating to disease severity (49). In patients with ovarian carcinoma, the host response to the tumor has been shown to be inhibited by Foxp3^+^CCR4^+^ Tregs recruited to the tumor by chemokines including CCL22 (50) and the CCR4-CCL22 axis appears to be operational in tolerized cardiac allografts (51). In our study, the previously observed co-expression of CCR4 and CLA (29) raises the possibility that this subset of Tregs migrate into the skin where they accumulate over years and dampen immune surveillance and contribute to tumor escape, hence subsequent cuSCC development. Based on infection models, the majority of CCR4^+^ lymphocytes are in an activated state and appear to exert suppressive functions by secreting a number of immunosuppressive cytokines (52).

Participants enrolled in the first year of the VIRUSCAN Study and included in this analysis were followed for up to 4 years. The incidence plots suggest that the magnitude of the association between baseline activated CCR4^hi^ Tregs in circulation and subsequent cuSCC may increase over time. However, it is unclear whether CCR4^hi^ Tregs are driving the effect on cuSCC independently, or in combination with CLA^+^ Treg. As previously described, CCR4^hi^ Tregs and CLA^+^ Tregs are strongly correlated (29) and few study participants had lower CCR4^hi^ Treg levels but higher CLA^+^ Treg levels. Thus, teasing apart the effect of CLA^+^ Treg from CCR4^hi^ Treg will require a larger sample size. Future expansion of the present analysis to all VIRUSCAN Study participants with extended follow-up time will allow us to further investigate the independent and joint effects of CCR4^hi^ Treg and CLA^+^ Treg on the development of cuSCC while examining potential differences in risk with increasing follow-up time. Furthermore, future studies are needed to validate the clinical utility of the Treg cut points defined here.

The present study is the first to investigate activated CCR4^hi^ Tregs in circulation as a predictor of cuSCC in the context of recent UVR. Our ability to identify proportional increases in CCR4^hi^ and Foxp3 expressing T cells in KC tissue in patients with evidence of increasing chronic UVR suggests they may play a direct role in exerting local immunosuppression in susceptible individuals. While cumulative UVR is a known risk factor for cuSCC and may be considered when determining the appropriateness and frequency of total body skin exams for skin cancer screening, a majority of individuals in the present study (82.9%) did not develop cuSCC during the follow-up period. Therefore, novel biomarkers of risk, such as distinct subsets of activated Tregs in circulation such as the described CCR4hi Tregs could be critically important for more accurate risk stratification of patients for cuSCC prevention and screening. Furthermore, a better understanding of the biological underpinnings of the observed associations between activated Tregs in circulation and cuSCC may lead to the development of novel prevention and/or treatment strategies. In this regard, anti-CCR4 antibodies have been evaluated as a potential strategy to remove CCR4-expressing Tregs from the tumor microenvironment to enhance effector T cell function. Such strategy could be explored for depletion of circulating CCR4^hi^ Treg populations in patients deemed high risk for cuSCC development (26).

## Data Availability Statement

The datasets presented in this article are not readily available in order to protect participant confidentiality and privacy. Requests to access the datasets should be directed to dana.rollison@moffitt.org.

## Ethics Statement

The studies involving human participants were reviewed and approved by University of South Florida Institutional Review Board. The patients/participants provided their written informed consent to participate in this study.

## Author Contributions

DER, PE-B, DA, MS, and JM contributed to the conception and design of the study. JM, RH, and AA carried out the experiments. BC and NF assisted in patient recruitment. YZ performed the statistical analysis. DER, YZ, RA, and PE-B wrote the original draft of the manuscript. All authors contributed to manuscript revision, read, and approved the submitted manuscript.

## Funding

This work was supported by the National Cancer Institute at the National Institutes of Health (Grant R01-CA177586) and a Moffitt Cancer Center Team Science grant. This work was also supported by the Flow Cytometry Core, Participant Research, Interventions, and Measurement (PRISM), Analytic Microscopy and Tissue Cores at the H. Lee Moffitt Cancer Center and Research Institute, a comprehensive cancer center designated by the National Cancer Institute and funded in part by Moffitt's Cancer Center Support Grant [P30-CA076292]. The funders were not involved in the study design, data collection/analysis, or manuscript preparation.

## Conflict of Interest

DER and PE-B filled a provisional Patent titled “Methods of detecting risk of a cancer,” application serial #63/041,676 filed on 6/19/2020. DER serves on the Board of Directors for NanoString Technologies, Inc. PE-B and the Moffitt Cancer Center and Research Institute received payment from licensing unrelated technology to Celgene Corporation and have an appropriate conflict-of-interest management plan. AA is currently employed by AstraZeneca. The remaining authors declare that the research was conducted in the absence of any commercial or financial relationships that could be construed as a potential conflict of interest.

## Publisher's Note

All claims expressed in this article are solely those of the authors and do not necessarily represent those of their affiliated organizations, or those of the publisher, the editors and the reviewers. Any product that may be evaluated in this article, or claim that may be made by its manufacturer, is not guaranteed or endorsed by the publisher.
