# Supplementary material for: Circulating Immunosuppressive Regulatory T Cells Predict Risk of Incident Cutaneous Squamous Cell Carcinoma
Source: Front Med (Lausanne). 2021 Nov 2;8:735585. doi: 10.3389/fmed.2021.735585 (PMC8593034; doi:10.3389/fmed.2021.735585)

Supplementary Material

**Supplementary Figure 1. Flow cytometry gating strategy for Treg cell subpopulations.** Doublet and non-viable cells were first excluded and PBMCs were gated on CD4 and CD3 (not shown). CD4 T cells were then gated according to Foxp3 and CD25 expression and further characterized by CLA, CCR4, CD27 and CD45RA expression. Gates were determined by fluorescence-minus-one controls (FMO). TEM, terminal effector memory; N, naïve; CM, central memory; E, effector.


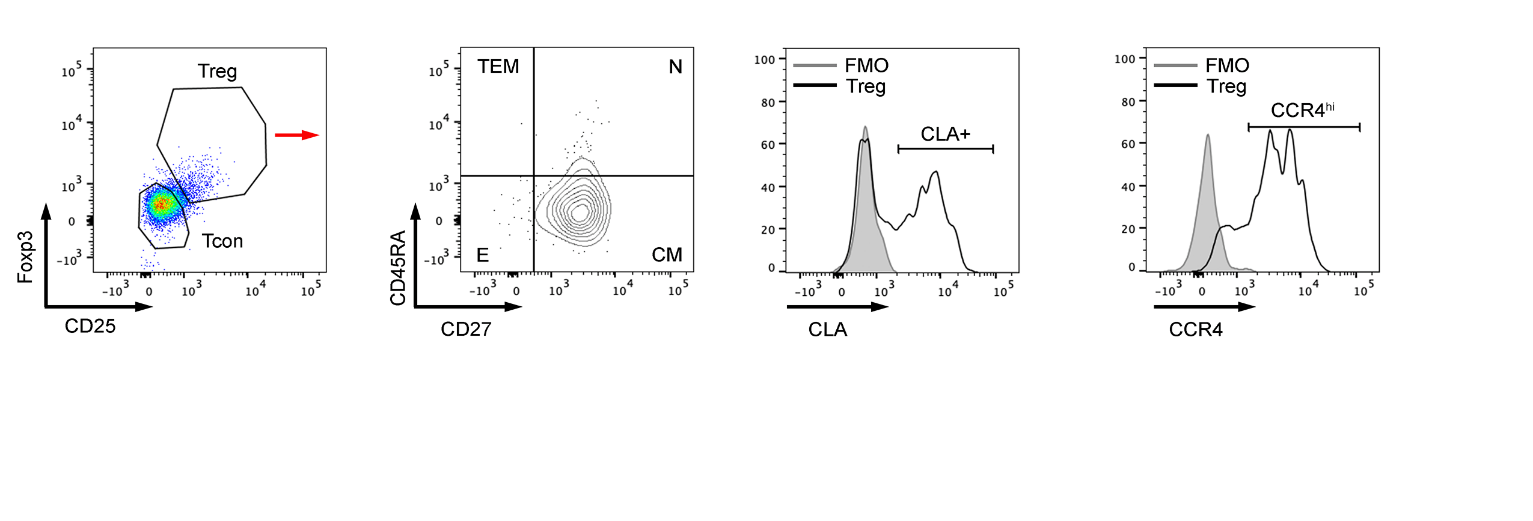


| **Supplementary Figure 2. Cumulative incidence for cutaneous squamous cell carcinoma (cuSCC) by baseline levels of circulating Treg cells in three groups.** The cumulative incidence for cuSCC stratified by levels of baseline circulating Treg, separately for CCR4^hi^ Treg (A), CLA^+^ Treg (B), and CD45RA^-^/CD27^-^ Treg cells (C), as determined by the reduced monotonic regression prior to regrouping. |
| --- |


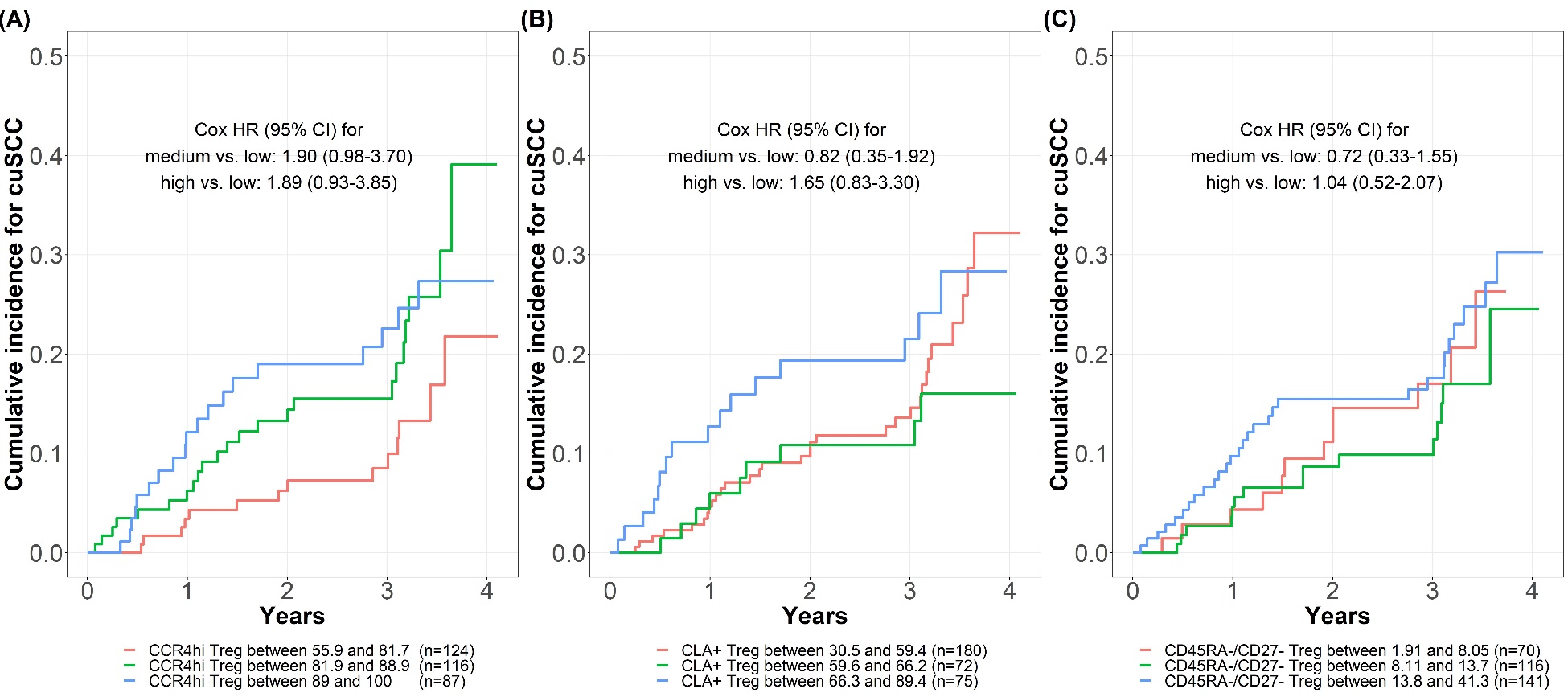


| **Supplementary Figure 3. Association between spectrophotometer-measured UV exposure (UVR) and incident cutaneous squamous cell carcinoma (cuSCC).** The cumulative incidence of cuSCC was plotted by levels of UVR. (**A**) UVR was categorized into tertiles and similar cuSCC incidence rates were observed between the 2^nd^ and 3^rd^ tertiles of UVR. (**B**) The 2^nd^ and 3^rd^ tertile of UVR were combined and compared to the lowest tertile of UVR and higher cuSCC incidence rates were observed in the higher UVR group as compared to the lower UVR group although this result did not reach statistical significance. |
| --- |

**
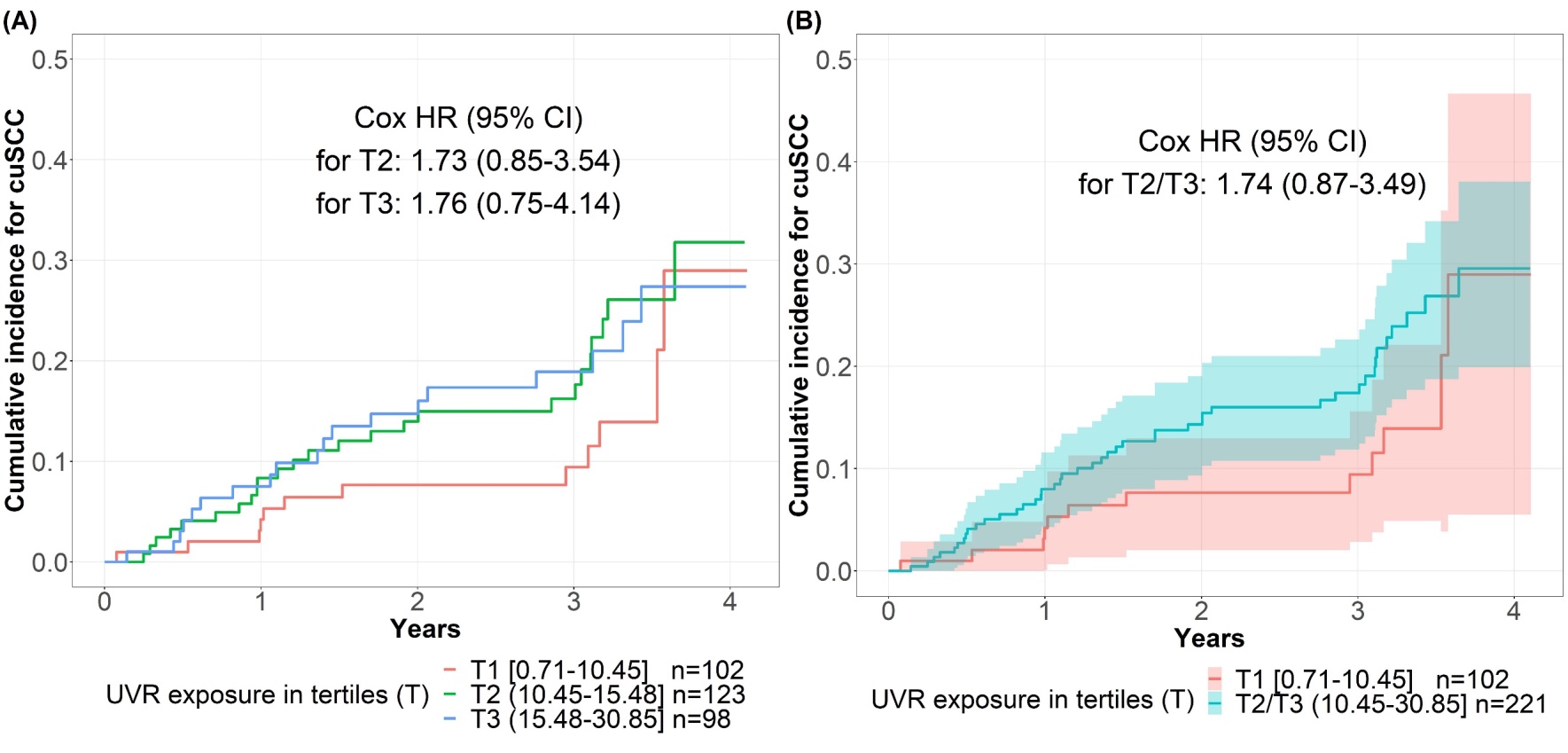
**

**Supplementary Figure 4. Illustrative examples of solar elastosis grading and corresponding immunohistochemistry (IHC) staining of Foxp3 (brown) and CCR4 (red) staining in incident cutaneous squamous cell carcinoma (cuSCC) tumors, magnification 10x.** Panels A-C depict perilesional skin of cuSCC tumors with solar elastosis grading of 1, 2, and 3, respectively. Panels D-F depict the corresponding IHC staining for CCR4 and FOXP3, demonstrating increasing levels of CCR4 and FOXP3 in skin with higher levels of solar elastosis. The arrows in D-F point to the areas of greatest inflammation within each tumor. Greater degrees of solar elastosis may increase distance from infiltrate to epidermis.


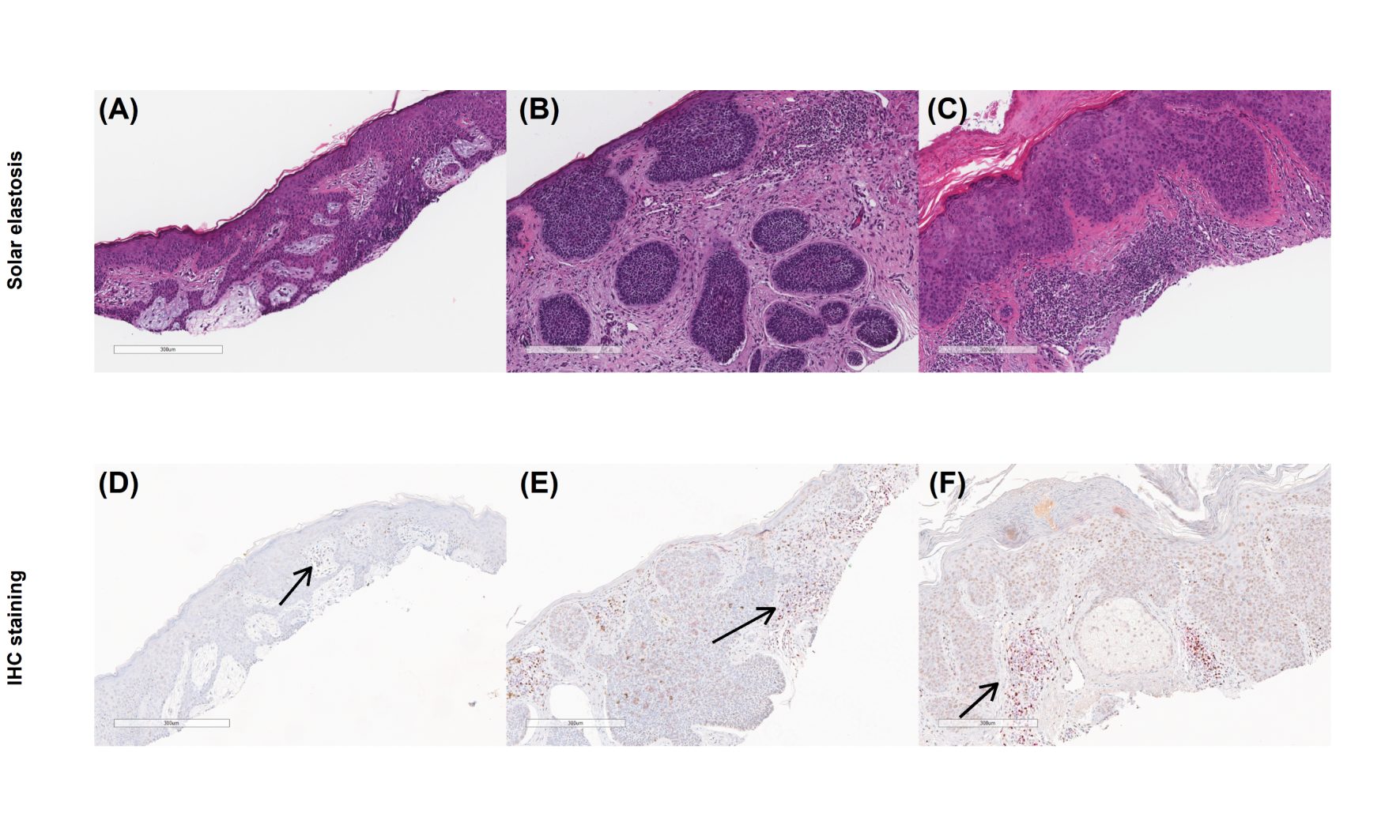

Supplement: Supplementary file 1 [file Data_Sheet_1.docx]
